# Alcohol Septal Ablation for Hypertrophic Obstructive Cardiomyopathy

**DOI:** 10.2174/157340308785160561

**Published:** 2008-08

**Authors:** Hicham El Masry, Jeffrey A Breall

**Affiliations:** Krannert Institute of Cardiology, Indiana University School of Medicine

**Keywords:** Hypertrophic cardiomyopathy, left ventricular outflow obstruction, alcohol ablation.

## Abstract

Since its original description in 1994, alcohol septal ablation (ASA) has emerged as a minimally invasive modality for treatment of hypertrophic obstructive cardiomyopathy compared to surgical myomectomy. This catheter-based intervention relies on the injection of absolute alcohol into the septal perforator to induce a controlled infarction of the hypertrophied septum and consequently abolish the dynamic outflow obstruction. This gradient reduction has been correlated with a significant clinical improvement in the patient’s symptomatology and with left ventricular remodeling. The procedure has been refined throughout the years, especially with the introduction of myocardial contrast echocardiography for localization of the area at risk of infarction and the reduction in the amount of alcohol used. Major complications of ASA are uncommon in large referral centers but conduction system disturbances has been the most commonly reported complications of ASA with 10% of patients necessitating permanent pacemaker implantation for complete heart block. ASA has not been compared to the gold standard surgical myomectomy in a randomized prospective study. We review the clinical aspects of this procedure and provide some historical background.

## INTRODUCTION

Hypertrophic cardiomyopathy (HCM) is a genetic cadiomyopathy inherited as an autosomal dominant trait and characterized by asymmetrical septal hypertrophy unexplained by any other cardiac disease. Microscopically, this disease process is notable for a distinct abnormal disorderly array of myofiber orientation. The disease has a wide spectrum of clinical presentation varying from totally asymptomatic individuals with normal life expectancy to severely symptomatic patients with increased mortality due to sudden death or congestive heart failure. The overall prognosis of HCM patients is favorable with an annual mortality of about 1% and adverse events are mostly related to sudden cardiac death, obstructive symtoms or progressive heart failure. Left ventricular outflow obstruction is present in 25-30% of patients with HCM and constitutes the pathophysiological basis for many aspects of the disease. It is also an important prognostic factor of the disease. In fact, it has been clearly shown that a gradient of 30 mmHg or more is associated with an increased risk of disease progression, congestive heart failure, stroke and death. While the treatment of obstructive HCM has largely relied on pharmacologic management of the disease with beta-blockers and calcium channel blockers, a small but important subgroup of patients are refractory to medical therapy. Surgical myectomy and percutaneous alcohol septal ablation have proven efficacy in the symptomatic management of this drug-refractory population. Alcohol septal ablation was developed as a less invasive approach to reduce left ventricular outflow obstruction by inducing a partially controlled infarct of the basal septum with resultant scar formation and left ventricular remodeling.

## INDICATIONS FOR SEPTAL REDUCTION

Septal reduction therapies are largely reserved for the subgroup of patients with obstructive HCM who continue to experience significant symptomatology despite maximized medical therapy: limited functional capacity secondary to exertional dyspnea and chest pain classified as NYHA III-IV or CCS III-IV. In addition, the ACC/ESC recommends that two additional selection criteria be employed in selecting patients for this procedure 1) septal hypertrophy of 18mm or more and 2) resting or provocable gradient of 50 mmHg or more [1]. However, with the increased experience and relative safety of ASA, these criteria have been relaxed and the procedure is now used to treat an increasing number of patients with obstructive symptoms. In fact, in a recent systematic review of almost 3000 patients undergoing ASA by Alam *et al*., the mean NYHA class was 2.9 (±0.01), and only 51-53% percent were on beta-blocker or calcium channel blocker therapy [2].

The preprocedural evaluation of candidates for ASA should include a rigorous clinical and echocardiographic investigation of comorbidities and other cardiac anomalies. A careful review of the patients’ medical therapy should be undertaken to assess the potential for symptomatic improvement by optimization of this regimen. Moreover, an evaluation for concomitant abnormalities which warrant definitive surgical therapy is necessary. These abnormalities include discrete subaortic stenosis, severe coronary artery disease and structural abnormalities of the mitral valve. In particular mitral valvular apparatus abnormalities are common in these patients (7-10%) and include: abnormal insertion of the papillary muscle into the anterior mitral valve leaflet and abnormal fibrous attachment or fusion of the papillary muscles to the septum or LV free wall both of which result in midcavitary fixed obstruction [3, 4]. Careful echocardiographic evaluation of the mitral regurgitant jet timing and direction is usually helpful in excluding these pathologies. 

## PROCEDURE DETAILS

Percutaneous alcohol septal ablation was introduced in 1994 as a less invasive alternative to surgical myomectomy and was originally targeted to a population of symptomatic patients who were thought to be poor surgical candidates. The procedure has undergone significant technical refinements most notable of which is the introduction of myocardial contrast echocardiographic localization of the target area. 

After obtaining arterial access, the procedure is usually initiated with a reevaluation of the patient’s hemodynamics and re-measurement of the intracavitary gradient. This is performed through simultaneous pressure measurement in the left ventricle with an end-hole catheter and the aorta. Physiologic provocation of the gradient is then assessed with the Valsalva maneuver and checking for the Brockenbrough sign by inducing a ventricular extrasystole. Venous access, usually through the femoral vein, is also obtained and a temporary transvenous pacemaker is inserted into the RV apex. Coronary angiography is then performed to exclude severe coronary disease and to locate the first septal perforator. Usually this vessel arises from the proximal LAD, however it might have a separate takeoff from the left main, or branch from the left circumflex, ramus intermedius, or the right coronary. Uncommonly, a distinct first septal perforator cannot be located which obviates the procedure’s feasibility. The septal perforator is then engaged with an over-the-wire balloon, the lumen of which serves to inject contrast and alcohol. The balloon is then inflated and a selective angiogram is performed to exclude its encroachment onto the LAD. Angiographic contrast may also be injected through the balloon lumen to ensure no spill of the contrast back into the LAD. During inflation, a continuous monitoring of the gradient might reveal a significant reduction indicating a favorable target vessel and usually portends a good response to ASA [4]. 

Due to anatomical variation of the septal perforator, the area supplied by this vessel might include a variable amount of myocardial tissue that might not only involve the hypertrophied basal septum but also the right ventricular septum and even the LV apex [5]. In that respect, evaluation of the area at risk by myocardial contrast echocardiography is considered a key aspect for the localization of the ablation which ideally should only be localized to the proximal septum at the point of contact with the anterior mitral leaflet. Echocardiographic contrast is injected thru the balloon and intraprocedural two-dimensional transthoracic echocardiogram is performed to visualize the perfusion area prior to alcohol injection [6, 7]. Patients with a large area at risk or who have a significant involvement of the right ventricle have a higher complications rate including complete atrioventricular block [7, 8]. In cases where the septal perforator supplies large territories, lower doses of alcohol may be used. Also, if the vessel bifurcates, the branch supplying the basal septum should be used.

After localizing the target vessel, the transvenous pacemaker is checked for capture and intravenous analgesia is administered. Then a slow injection of absolute alcohol is performed through the target vessel and a gradient reassessment is performed to demonstrate an initial partial response defined. After deflation of the balloon, a final angiogram is performed to ensure the patency of the LAD (this usually shows an occluded septal perforator at the site of injection).

The dose as well as the rate of alcohol injection during ASA have been the subject of continuous reevaluation and vary between institutions. Original studies used 3-4ml injections however in a recent randomized controlled trial of thirty four patients undergoing ASA, a lower dose (less than 2 ml) of alcohol injected resulted in similar reductions in the outflow gradient and symptomatic improvement at six-month follow up [9]. The amount of alcohol injected has been correlated with the area of necrosis (peak CKMB) and has been shown to be an independent predictor of ASA complications and post-procedural mortality [10]. Moreover a slower rate of alcohol injection has been correlated with a decreased incidence of complete heart block in recent series [4, 11, 12].

## STRUCTURAL REMODELING AND HEMODYNAMIC EFFECTS OF ASA

Although ASA is followed by an immediate decrease of the outflow gradient, most of the acute diminution likely represents stunning and is pathophysiologically different from the more delayed phenomenon which is a result of akinesis and thining of the basal septum. The immediate abolition of the outflow gradient may be followed by a recurrence of the gradient 1 to 3 days after ASA. Then, a permanent and more substantial reduction in the gradient (mean LVOT gradient from 65 to 31 mmHg [2]) progressively develops within 3 to 12 months after the procedure and is a direct result of scar formation, thinning, and left ventricular remodeling [13-15]. ASA has been also associated with a significant decrease and even abolition of mitral regurgitation on long term follow-up [16].Cardiac magnetic resonance imaging (CMR) and strain rate imaging have been used to evaluate the effect of remodeling on the LV systolic function. These studies showed a significant decrease in myocardial mass and LV wall thickness both in the infarcted and non infarcted areas at early and midterm follow up after ASA [17-19]. Moreover, a small decrease in the ejection fraction is noted in these follow up studies with a paradoxical increase in global systolic indices (shortening index and Tei index) [17, 20]. Diastolic function has been extensively studied in patients with HCM and follow up studies of post-ASA patients have shown a significant and sustained improvement in echocardiographic diastolic parameters [20, 21]. In a recent study by Jassal *et al.*, all patients had a pseudonormal or an impaired relaxation pattern at baseline along with left atrial dilation. They showed significant improvement in E-wave deceleration time, isovolumic relaxation time, early diastolic mitral lateral annular velocity (*E*'), mitral inflow propagation velocity, ratio of transmitral early LV filling velocity (*E*) to early diastolic Doppler tissue imaging of the mitral annulus (*E*/*E*'), and *E*/*V*_p_ were observed on long term follow up one and two years following successful ASA [22].

## CLINICAL OUTCOMES AND POTENTIAL COMPLICATIONS AFTER ASA

ASA has been associated with a favorable clinical response in short and intermediate-term follow-up studies. In a recent systematic review of 42 studies involving 2959 patients, Alam *et al.* demonstrated a significant improvement in heart failure symptoms with mean NYHA class from 2.9 to 1.2 as well as angina with mean CCS class from 1.9 to 0.4 at 1 year follow-up [2]. Peak oxygen consumption increased from 17.8 to 23.6 ml/kg per minute and mean exercise capacity on a treadmill from 325.3 to 437.5 seconds [2].

Complications of ASA, although rare in high volume centers, occur in the early post-procedural period and include LAD dissection, coronary artery spasm, cardiac tamponade, cardiogenic shock, pulmonary embolism and stroke. Most commonly, however, ASA causes a transient or permanent disruption of the conduction system: first degree AV block develops in 53% of patients while RBBB block in 46% and complete heart block (CHB) requiring permanent pacemaker (PPM) implantation in 10.5% of patients. Interestingly, CHB may be a transient phenomenon in 10 to 46% of patients with recovery within the first 24 hours [23, 24], while few patients might develop CHB up to nine days after ASA [11, 25]. This may have an important impact on post-procedural care. Specifically the length of hospital stay that varies according to institutional protocol from at least 24-48 hours up to five days of CCU monitoring [4, 24, 26]. Several studies evaluated determinants of complete heart block after ASA in an effort to identify a high risk group that would benefit from a PPM implantation prior to the procedure. While age >55, female gender, bolus injection of alcohol, injection of more than one septal perforator, volume of alcohol injection and lack of use myocardial contrast echocardiographic localization are all potential factors, only the presence of LBBB on baseline ECG has been persistently identified as a strong predictor of CHB [11, 23, 27, 28]. Faber *et al.* proposed a scoring system based on electrocardiographic markers of abnormal AV conduction (PQ interval, QRS duration and baseline heart rate), the severity of the LVOT gradient, reversibility of CHB, and timing of the SGOT peak. Based on this scoring system, three risk groups could be identified: low risk patients can be discharged after short term monitoring (48 hours), a high risk group where an elective PPM implantation should be considered, and an intermediate group for whom prolonged monitoring and expectant management are warranted [24]. Based on the present knowledge, it is still unclear if prophylactic PPM implantation is indicated for patients with LBBB at baseline and larger scale prospective evaluation is needed to answer this question. 

Ventricular arrhythmias have been reported and include ventricular fibrillation (2.2%) [2] and less commonly sustained ventricular tachycardia (VT) [29-32]. Typically, they develop in the early post-procedural period and are self-limited but few patients present with VT up to 3 weeks after ASA. The mechanism of these arrhythmias is controversial and has been suggested to be secondary to reentry around the MI-induced scar; however the arrhythmogenic potential of the myofibrillar disarray characteristic of HCM can’t be excluded. All patients reported in the literature with sustained VT after ASA underwent ICD implantation hence adding a therapeutic challenge to the management of these patients. 

Early mortality occurring within the first 30 days after ASA has been reported to be 1.5% (0-5%) and results usually from hemodynamic collapse due to ventricular failure or tamponade, ventricular fibrillation or LAD dissection [2]. Unfortunately, few reports address the long term safety and outcomes of ASA especially late all-cause mortality. Kuhn *et al.* reported on a single center experience with ASA where 644 patients were followed for up to 10 years. In their report, they described a significant decrease in the incidence of complications correlating with decreasing alcohol dosage (from a mean of 2.9 ml to 0.8 ml). In fact, in-hospital mortality decreased from 1.8% to 0.6% and was mostly ascribed to non-cardiac causes of death [10]. It is clear however that an improved expertise might be an integral part in the refinement of the procedure with subsequent decrease of the complication rate. After hospital discharge, limited data indicates an annual mortality rate of 3.2% which is comparable to the natural history of high-risk HOCM patients. 

## COMPARISON OF ASA AND SEPTAL MYECTOMY

Since its introduction by Sigwart in 1994, ASA has been performed in thousands of patients with good short term results. Acute anatomical changes after ASA and myomectomy have been evaluated by cardiac MRI; ASA results in a larger area of tissue necrosis (16±7 grams compared to 6±4 grams) often involving a transmural infarct with varying location and depth at the junction of the anterior and inferior segments of the basal septum [33]. Some patients have had extension of the infarction into the right ventricular side of the septum with associated residual gradient on follow-up and less favorable symptomatic relief [34]. Myomectomy results in a more localized resection of the LV aspect of the basal septum as compared to the deeper scar of ASA offering a potential explanation of the association of ASA with RBBB and of the myomectomy with LBBB. Aside of these morphological differences, myomectomy is associated with a lower risk of complete AV block necessitating permanent pacemaker implantation (5% *vs*. 17%) [35] and an immediate clinical benefit although at the cost of a higher risk of strokes and a longer recovery period postoperatively [36].

Few reports compared the clinical outcomes of ASA to surgical myomectomy and no comparison of long term outcomes has been performed. In a recent meta-analysis of three retrospective studies including 171 patients [37-40], both interventions had comparable effect on interventricular septal thickness reduction, left ventricular end-diastolic dimension increase and NYHA class improvement [35]. However, there was a small but significant difference in the relief of the LVOT gradient: from 76.0 to 15.7 mmHg in ASA group compared to a decrease from 74.7 to 9.4 mmHg in the myomectomy group (p<0.05) [35]. It is difficult to predict any role of this difference in affecting the clinical outcomes after ASA and this question needs to be evaluated prospectively with longer follow-up period. Moreover, all reports comparing ASA and myomectomy usually involved an older cohort with more comorbidities for patients receiving the percutaneous therapy and it remains unclear if these factors may interact with the treatment effect. 

## CONCULSIONS

ASA has developed as a safe and attractive modality to treat patients with HCM whose symptoms are refractory to optimal medical therapy with negative inotropic agents. The procedure has been refined over the past decade since its development with steadily improving outcomes and reduced complications especially PPM implantation. However, concerns over the higher risk of complete AV block and development of a potentially arrhythmogenic scar after ASA have been raised [41]. The gold standard for HCM has been classically considered the surgical approach that has been time-tested over the past forty years, although many interventional cardiologists consider ASA as a first line intervention due to its ease of performance and less invasive nature. Either procedure is best performed by an experienced operator. The feasibility of comparing both modalities in a randomized controlled trial has been questionned due to the low frequency of the disease and the low event rates after these interventions [42]. Alternatively, a multicenter prospective evaluation of non-randomized patients with long term follow-up would be reasonable to resolve this controversy. Meanwhile, ASA is a reasonable therapeutic option especially in older patients with significant comorbidities (especially lung disease) and a favorable coronary anatomy in the absence of mitral valve disease.

## Figures and Tables

**Fig. (1):**
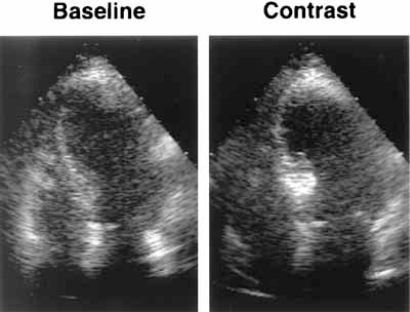
Apical four-chamber views illustrating the use of myocardial contrast echocardiography to determine area at risk during ASA: injection into the first septal perforator results in enhancement of the proximal basal septum. (adapted with permission from Nagueh, S. F., *et al.* J Am Coll Cardiol 1998; 32: 225-229).

**Fig. (2):**
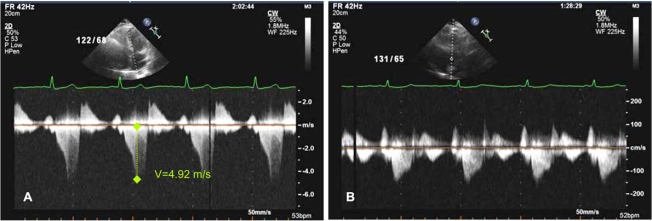
Transthoracic echocardiogram in hypertrophic obstructive cardiomyopathy: continous wave Doppler across the LVOT (A) before ASA indicating severe obstruction with a dynamic gradient of 96 mmHg and (B) 6 months after ASA with resolution of the obstruction.
